# Characterization of the complete plastid genome of lop-sided onion *Allium obliquum* L. (Amaryllidaceae)

**DOI:** 10.1080/23802359.2018.1456369

**Published:** 2018-03-26

**Authors:** Mikhail A. Filyushin, Alexey V. Beletsky, Alexander M. Mazur, Elena Z. Kochieva

**Affiliations:** a Institute of Bioengineering, Research Center of Biotechnology of the Russian Academy of Sciences, Moscow, Russia;; b Faculty of Biology, Lomonosov Moscow State University, Moscow, Russia

**Keywords:** *Allium obliquum*, lop-sided onion, chloroplast genome

## Abstract

The complete chloroplast genome sequence of *Allium obliquum* was determined by Illumina single-end sequencing. The complete plastid genome was 152,387 bp in length, containing a large single copy (LSC) of 81,588 bp and a small single copy (SSC) of 18,059 bp, which were separated by a pair of 26,370 bp inverted repeats (IRs). A total of 134 genes were annotated, including 83 protein coding genes, 38 tRNA genes, eight rRNA genes, and five pseudogenes. The overall GC contents of the plastid genome were 36.8%. Unlike *A. cepa* (onion) and *A. sativum* (garlic), *A. obliquum* encodes a functional intact *infA* gene.

Lop-sided onion, *Allium obliquum* L., is a perennial bulbous plant with wide geographic distribution in Eurasia from Romania to China and Mongolia. It is popular as an ornamental plant because of the spectacular yellow inflorescences (Seregin et al. 2015). Young leaves *A. obliquum* are used by local people as food in places of growth (Friesen 1988).

The plastid DNA *A. obliquum* (cultivar Novichok; seed from Federal Scientific Vegetable Center, Moscow oblast, Russia) was amplified via long range PCR using 11 pairs of primers developed on the basis of the *Allium cepa* plastid genome (KF728079, NC_024813), sequencing was conducted using the Illumina HiSeq 1500 Sequencing System with single-end 220 bp reads. Spades v.3.8 was used to assemble the high-quality short reads into contigs (Bankevich et al. 2012). Contigs were assembled against the complete *A. cepa* plastome as a reference (NC_024813). Gaps were closed using assembly graph in Bandage (Wick et al. 2015), reads were then mapped against the resulting single contig to ensure the correctness of the finished assembly. The plastid genome of *A. obliquum* was annotated by using the DOGMA program (http://dogma.ccbb.utexas.edu). The start and stop codons for the genes were identified and corrected manually. A circular plastid genome map of *A. obliquum* was drawn using the OGDRAW program (Lohse et al. 2013).

The assembled *A. obliquum* plastid genome (MG670111) was 152,387 bp in length, showing a typical quadripartite structure including a pair of inverted repeats (IRs) of 26,370 bp separating one large single copy (LSC) region of 81,588 bp and one small single copy (SSC) region of 18,059 bp. GC contents of the genome were 36.8%. A total of 134 genes were identified that include 83 protein-coding genes, 38 tRNA genes, eight rRNA genes, and five pseudogenes.

Most of the genes are single copy, whereas 18 genes present in double copies, including six protein-coding genes (*rps19*, *rpl2*, *rpl23*, *ycf2*, *ndhB*, and *rps7*), eight tRNA genes (*trnR-ACG*, *trnL-CAA*, *trnV-GAC*, *trnH-GUG*, *trnI-CAU*, *trnI-GAU*, *trnA-UGC*, and *trnN-GUU*), and all four rRNA genes in IRs (*rrn4.5*, *rrn5*, *rrn16*, and *rrn23*). All genes had a common start codon (ATG) in the initiation site, except *rps19*, which carried GTG as a start codon. Intron sequences are found in 17 genes, 15 of which contain a single intron (*atpF*, *rpoC1*, *ndhA*, *trnK-UUU*, *trnG-GCC*, *trnL-UAA*, and *trnV-UAC*; four genes in IRs: *rpl2*, *ndhB*, *trnI-GAU*, and *trnA-UGC*), while two (*clpP* and *ycf3*) have two introns.

Five genes became pseudogenes due to internal stop codons (*rps2*, two *ycf15* in IRs) or because of incomplete duplication in the IRB/SSC junction region (*ycf1*) and the exon II deletion of the *rps16* gene (verified by additional sequencing). *Allium obliquum* encodes a functional intact *infA* gene unlike *A. cepa* (NC_024813; von Kohn et al. 2013) and *A. sativum* (NC_031829; Filyushin et al. 2016) where *infA* was found to be a pseudogene.

The ML tree was clearly divided into two clades with the order level, Asparagales and Liliales. *Allium obliquum* is clustered with other sampled *Allium* species with 100% bootstrap values (Figure 1).

**Figure 1.**
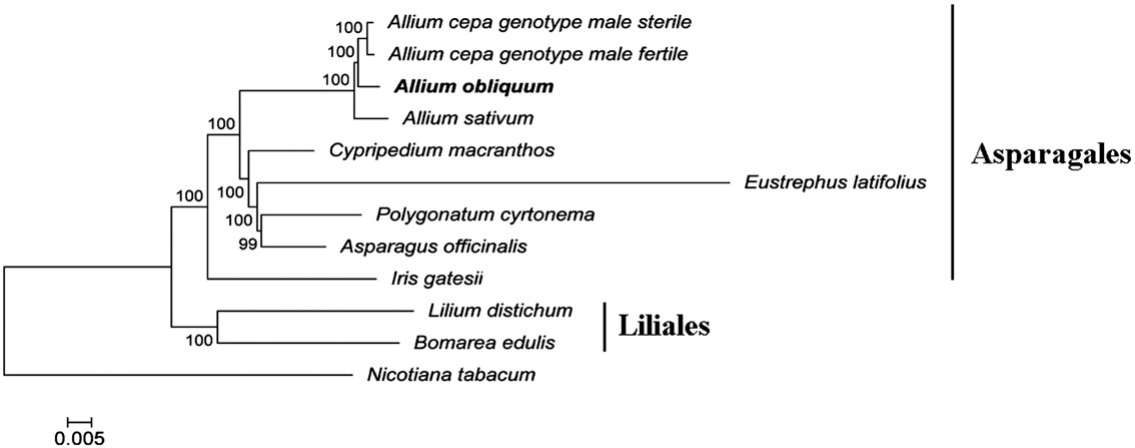
Phylogenetic tree inferred by maximum-likelihood using 82 protein-coding gene sequences of 10 species including seven species from the Asparagales order: *Allium cepa* (genotype male sterile KF728079 and genotype male fertile NC_024813), *Allium sativum* (NC_031829), *Eustrephus latifolius* (KM233639), *Polygonatum cyrtonema* (KT630835), *Cypripedium macranthos* (KF925434), *Asparagus officinalis* (NC_034777), *Iris gatesii* (KM014691); two species from Liliales order: *Bomarea edulis* (NC_025306), *Lilium distichum* (NC_029937); and *Nicotiana tabacum* (NC_001879) as an outgroup. PhyML 3.1 (Guindon et al. 2010) was used for the sequence alignment and construction of the tree. Bootstrap support values based on 1000 replicates are displayed on each node.
